# Evaluation of Low-Value Diagnostic Testing for 4 Common Conditions in the Veterans Health Administration

**DOI:** 10.1001/jamanetworkopen.2020.16445

**Published:** 2020-09-22

**Authors:** Thomas R. Radomski, Robert Feldman, Yan Huang, Florentina E. Sileanu, Carolyn T. Thorpe, Joshua M. Thorpe, Michael J. Fine, Walid F. Gellad

**Affiliations:** 1 Division of General Internal Medicine, Department of Medicine, University of Pittsburgh School of Medicine, Pittsburgh, Pennsylvania; 2 Center for Health Equity Research and Promotion, Veterans Affairs Pittsburgh Healthcare System, Pittsburgh, Pennsylvania; 3 UPMC Center for High-Value Health Care, UPMC Insurance Services Division Steel Tower, Pittsburgh, Pennsylvania; 4 Division of Pharmaceutical Outcomes and Policy, Eshelman School of Pharmacy, University of North Carolina, Chapel Hill

## Abstract

**Importance:**

Low-value care is associated with harm among patients and with wasteful health care spending but has not been well characterized in the Veterans Health Administration.

**Objectives:**

To characterize the frequency of and variation in low-value diagnostic testing for 4 common conditions at Veterans Affairs medical centers (VAMCs) and to examine the correlation between receipt of low-value testing for each condition.

**Design, Setting, and Participants:**

This retrospective cohort study used Veterans Health Administration data from 127 VAMCs from fiscal years 2014 to 2015. Data were analyzed from April 2018 to March 2020.

**Exposures:**

Continuous enrollment in Veterans Health Administration during fiscal year 2015.

**Main Outcomes and Measures:**

Receipt of low-value testing for low back pain, headache, syncope, and sinusitis. For each condition, sensitive and specific criteria were used to evaluate the overall frequency and range of low-value testing, adjusting for sociodemographic and VAMC characteristics. VAMC-level variation was calculated using median adjusted odds ratios. The Pearson correlation coefficient was used to evaluate the degree of correlation between low-value testing for each condition at the VAMC level.

**Results:**

Among 1 022 987 veterans, the mean (SD) age was 60 (16) years, 1 008 336 (92.4%) were male, and 761 485 (69.8%) were non-Hispanic White. A total of 343 024 veterans (31.4%) were diagnosed with low back pain, 79 176 (7.3%) with headache, 23 776 (2.2%) with syncope, and 52 889 (4.8%) with sinusitis. With the sensitive criteria, overall and VAMC-level low-value testing frequency varied substantially across conditions: 4.6% (range, 2.7%-10.1%) for sinusitis, 12.8% (range, 8.6%-22.6%) for headache, 18.2% (range, 10.9%-24.6%) for low back pain, and 20.1% (range, 16.3%-27.7%) for syncope. With the specific criteria, the overall frequency of low-value testing across VAMCs was 2.4% (range, 1.3%-5.1%) for sinusitis, 8.6% (range, 6.2%-14.6%) for headache, 5.6% (range, 3.6%-7.7%) for low back pain, and 13.3% (range, 11.3%-16.8%) for syncope. The median adjusted odds ratio ranged from 1.21 for low back pain to 1.40 for sinusitis. At the VAMC level, low-value testing was most strongly correlated for syncope and headache (ρ = 0.56; *P* < .001) and low back pain and headache (ρ = 0.48; *P* < .001).

**Conclusions and Relevance:**

In this cohort study, low-value diagnostic testing was common, varied substantially across VAMCs, and was correlated between veterans’ receipt of different low-value tests at the VAMC level. The findings suggest a need to address low-value diagnostic testing, even in integrated health systems, with robust utilization management practices.

## Key Points

**Question:**

What is the frequency and degree of variation in low-value diagnostic testing for low back pain, headache, syncope, and sinusitis in the Veterans Health Administration?

**Findings:**

In this cohort study of 1 022 987 US veterans, low-value testing was performed for 5% to 21% of veterans with 1 of the 4 medical conditions. There was substantial variation in low-value testing across Veterans Affairs medical centers and significant correlation at the Veterans Affairs medical center level in veterans’ receipt of such testing.

**Meaning:**

In this study, low-value diagnostic testing was common and delivered variably throughout the Veterans Health Administration, suggesting the need to address the delivery of such care, even in integrated health systems, with robust decision support and utilization management.

## Teaser

This cohort study assesses the frequency and variation in low-value diagnostic testing for low back pain, headache, syncope, and sinusitis in the Veterans Health Administration.

## Introduction

Wasteful health care spending accounts for up to $935 billion, or 25% of total health care expenditures in the US.^1,2^ Over $100 billion has been spent on low-value care, defined as the use of health services for which immediate or downstream harms or costs exceed the potential benefits.^1,2^ Examples include cancer screening for patients with a limited life span or performing preoperative electrocardiography for patients undergoing low-risk cataract surgery.^3,4^ Up to 43% of Medicare beneficiaries have received low-value care, which may be associated with physical and psychological harm and an erosion of their trust in the health care system.^5,6^

Although the delivery of multiple specific low-value health services has been described among Medicare and private insurance beneficiaries, the use of such services in the Veterans Health Administration (VHA) has not been well characterized.^5,7,8,9,10,11,12^ As the largest integrated and federally operated health care system in the US, the practice environment at the VHA differs considerably from other systems, potentially impacting the provision of low-value care.^13^ For example, VHA clinicians do not operate within a fee-for-service system and are insulated from malpractice litigation. Moreover, the VHA electronic medical record contains information on veterans’ care across all Veterans Affairs Medical Centers (VAMCs) and decision support tools for ordering appropriate health services, which may be associated with a decreased likelihood of VA clinicians delivering low-value care.^14,15^ Nevertheless, several studies focused on the use of individual low-value health services (eg, prostate cancer screening and colonoscopy) have demonstrated that the delivery of low-value care still occurs at VAMCs.^3,16,17,18,19,20,21,22^

As health care costs continue to increase and recent legislation provides veterans with increasing flexibility regarding whether they receive care within or outside the VHA, quantifying the frequency and patterns of low-value care delivery is essential for the VHA to reduce wasteful health care spending without compromising quality or patient satisfaction.^15,23^ Our objective was to characterize the frequency of and VAMC-level variation in the delivery of low-value care, with a focus on low-value diagnostic testing for 4 common conditions in the VHA. We also sought to examine the degree of correlation among the receipt of different types of low-value diagnostic tests for each condition at the VAMC level.

## Methods

### Study Cohort and Data Sources

We conducted a retrospective cohort study of a 20% national random sample of veterans who were continuously enrolled in the VHA in fiscal year 2015. Veterans were required to have at least 1 outpatient face-to-face encounter with a VA clinician in this study year to ensure that they were actively receiving care at a VAMC. We excluded nonveterans, veterans younger than 18 years, and veterans treated in non-US facilities because they are not representative of the overall VA population.^18,22^ This cohort study was approved by the VA Pittsburgh Healthcare System institutional review board. Because deidentified administrative data were used, a waiver of informed consent was granted by this institutional review board. This study followed the Strengthening the Reporting of Observational Studies in Epidemiology (STROBE) reporting guideline.

We used the VA Enrollment Data File to identify veterans continuously enrolled in the VHA in fiscal year 2015 and the VHA Corporate Data Warehouse, which is a data repository, to identify veterans’ sociodemographic characteristics, medical comorbidities, and *International Classification of Diseases, Ninth Revision* and *Common Procedural Terminology* codes associated with each low-value test. We used the Planning System and Support Group file to characterize each veterans’ travel time to the nearest VA facility and the following sources to identify VAMC-level characteristics: outpatient visit volume (VA Corporate Data Warehouse), training status (VHA Allocation Resource Center), VAMC Complexity Score (VHA Office of Planning), and census region (US Census).

### Identification of Low-Value Diagnostic Testing

We applied a claims-based approach originally developed for use in Medicare data to identify low-value diagnostic testing in the VHA for 4 conditions that are common among VHA beneficiaries: nonspecific low back pain, uncomplicated headache, syncope, and acute sinusitis (Table 1 gives definitions of low-value testing for each condition).^5^ We chose these conditions and their related low-value tests because, for each condition, low-value testing is prevalent outside the VHA, the respective low-value tests are widely available at VAMCs, and the use of each low-value test is readily identifiable using VHA data (eAppendix in the Supplement).

**Table 1.** Definitions of Low-Value Diagnostic Testing for 4 Common Conditions Among Veterans

Medical condition	Sensitive criteria	Specific criteria^a^
Nonspecific low back pain	Back imaging with a low back pain diagnosis coded within 6 wk before imaging	Red flag symptoms coded within 30 d before imaging or undergoing imaging greater than 6 wk after the first diagnosis of low back pain^b^
Uncomplicated headache		
Head imaging	Head imaging with a headache diagnosis coded within 30 d before imaging	Red flag symptoms coded within 30 d before imaging^b^
Electroencephalography	Electroencephalography with a headache diagnosis coded within 30 d before testing	History of epilepsy or convulsions
Syncope		
Head imaging	Head imaging with a syncope diagnosis coded within 30 d before imaging	Red flag symptoms coded within 30 d before imaging^b^
Carotid ultrasonography	Carotid ultrasonography with a syncope diagnosis coded within 30 d before imaging	Diagnosis of stroke, transient ischemic attack or focal neurologic symptoms coded within 30 d before testing
Acute sinusitis	Computed tomography of the sinuses with a diagnosis of sinusitis coded within 30 d before imaging	Diagnosis of an immune disorder, nasal polyp, eyelid or orbital inflammation, or head or face trauma coded within 30 d before imagine or receipt of a diagnosis of sinusitis 30-365 d before testing

^a^ Specific criteria represent additional exclusions applied to veterans in the numerator for each condition.

^b^ eAppendix in the Supplement gives a listing of red flag conditions for each disease state.

Because a single set of criteria to identify low-value testing for each condition may not account for the value of a health service in each unique clinical situation, we applied both sensitive and specific criteria to identify the use of low-value testing.^5,7,18^ For example, to identify low-value testing (ie, unnecessary imaging) for low back pain, the sensitive criteria characterized any veteran who received low back imaging less than 6 weeks after receiving a low back pain diagnosis as having received low-value testing. This criterion was used because most cases of musculoskeletal low back pain resolve with conservative treatment within 6 weeks of onset. In acknowledging that testing may have been appropriate for some of these patients, the specific criteria further excluded veterans from the numerator who received testing with a diagnosis code for an emergent condition in the 30 days before undergoing testing (eg, cancer or neurologic impairment) and veterans who underwent their first test more than 6 weeks after their initial back pain diagnosis. This approach allowed us to account for variations in the literature regarding the characteristics that make a diagnostic test low value, establish a range of potential low-value testing use, and establish relative sensitivity and specificity for each criteria in the absence of an established gold standard.^5,7,18^

### Covariates

We established variables for age, sex, race/ethnicity, marital status, driving time to the nearest VAMC, Gagne Comorbidity Index, and VA priority group, which is assigned at the time of VHA enrollment and determines a veteran’s copay status for certain services. The Gagne Comorbidity Index (risk stratifies patients with score categories ranging from <0 to >9, with increased scores corresponding to increased risk of 1-year mortality) integrates elements of both the Charlson and Elixhauser Comorbidity indexes and has greater prognostic accuracy in predicting 1-year mortality.^24^ We used fiscal year 2014 data to generate these variables because this provided us with a fixed baseline of covariate values and we sought to capture low-value testing beginning in fiscal year 2015.

We assigned veterans to the 127 VAMCs nationwide where they received the majority of their outpatient care.^18^ We also identified each VAMCs’ US census region (eg, Northeast, Midwest, South, and West), size using outpatient visit volume, academic affiliation, and complexity score. The complexity score has 5 categories (1a, 1b, 1c, 2, and 3), which are determined using overall patient volume, patient case mix, number and nature of staff physician specialists, and intensive care unit capabilities; VAMCs classified as 1a are the most complex.^25^

### Statistical Analysis

Data analysis was performed from April 2018 to March 2020. We calculated the means and SDs for all continuous variables and frequencies for all categorical variables. From the overall cohort, we determined the number of veterans who were eligible to receive each low-value test based on their receipt of an *International Classification of Diseases, Ninth Revision* diagnosis code that corresponded to 1 of the 4 conditions of interest in fiscal year 2014 or before receipt of the related low-value test in fiscal year 2015. These subcohorts of eligible veterans were the denominators for each low-value testing measurement. Applying both the sensitive and specific criteria, we then determined the overall number (ie, numerator) of veterans who underwent low-value testing for each condition and the proportion of eligible veterans from each VAMC. Using mixed-effects logistic regression modeling, we determined the adjusted VAMC rates of low-value testing, which reflect the rates of low-value testing at each VAMC after removing the effects of that VAMC’s unique veteran- and VAMC-level characteristics. We also included random effects for each veteran’s assigned VAMC in our models.^26^ We conducted a planned subgroup analysis to assess the receipt of low-value testing in veterans at greatest risk of 1-year mortality and thus least likely to potentially benefit from such care, defined using the top decile of Gagne scores in the overall cohort.

We characterized variation in the delivery of low-value testing across VAMCs in 3 ways. First, to depict overall variation, we calculated the median adjusted odds ratio (aOR) of low-value testing for each condition. The median aOR characterizes the median odds of a veteran receiving a low-value test if 2 of 127 VAMCs are chosen at random and compared.^26^ Second, to characterize the proportion of variance in veterans’ receipt of low-value testing associated with variation between VAMCs, we calculated the intraclass correlation coefficient (ICC).^27,28^ We first calculated the ICCs using unadjusted models followed by models containing only veteran-level covariates and then both veteran- and VAMC-level covariates to assess the contribution of these variables to the between-VAMC variance. Third, to characterize variation across VAMCs with a greater degree of granularity, we divided VAMCs into deciles based on their frequency of low-value testing for each condition and compared the odds of low-value testing for each condition for veterans in each of the 9 higher deciles compared with the lowest decile.

At the VAMC level, we used the Pearson correlation coefficient to evaluate the degree of correlation between the delivery of low-value testing for each separate condition at VAMCs. We also determined the correlation between use of separate low-value tests for syncope (ie, head imaging and carotid ultrasonography) and uncomplicated headache (ie, head imaging and Electroencephalography) based on the algorithms available to identify specific types of testing for each of these conditions in the applied metric. Analyses were performed using SAS, version 7.1 (SAS Institute). A 2-sided *P* < .05 was considered statistically significant.

## Results

There were 1 022 987 Veterans in the overall cohort. The mean (SD) age was 60 (16) years, 1 008 336 (92.4%) were male, and 761 485 (69.8%) were non-Hispanic white (Table 2). A total of 343 024 veterans (31.4%) were diagnosed with nonspecific low back pain, 79 176 (7.3%) with uncomplicated headache, 23 776 (2.2%) with syncope, and 52 889 (4.8%) with acute sinusitis.

**Table 2.** Patient and VAMC Characteristics Overall and by Condition^a^

Characteristic	Overall cohort (N = 1 091 249)	Low back pain (n = 343 024)	Syncope (n = 23 776)	Uncomplicated headache (n = 79 176)	Acute sinusitis (n = 52 889)
**Patients**					
Age, mean (SD), y	60.1 (16.4)	57 (15.7)	63 (14.5)	52 (16.2)	56 (14.5)
Male	1 008 336 (92.4)	311 672 (90.9)	22 078 (92.9)	66 939 (84.5)	45 214 (85.5)
Race/ethnicity					
Non-Hispanic White	761 485 (69.8)	232 889 (67.9)	17 022 (71.6)	50 320 (63.6)	37 505 (70.9)
Non-Hispanic Black	179 803 (16.5)	66 888 (19.5)	4508 (19.0)	18 273 (23.1)	9866 (18.7)
Hispanic	52 888 (4.9)	18 890 (5.5)	882 (3.7)	5171 (6.5)	2325 (4.4)
Other or missing	97 073 (8.9)	24 357 (7.1)	1364 (5.7)	5412 (6.8)	3193 (6.0)
Married	528 337 (48.4)	170841 (49.8)	11 400 (48.0)	36 542 (46.2)	26 661 (50.4)
VA priority group^b^					
1	282 703 (25.9)	124 285 (36.2)	8184 (34.4)	32 090 (40.5)	19 323 (36.5)
2	88 289 (8.1)	32 015 (9.3)	1617 (6.8)	7183 (9.1)	4690 (8.9)
3	134 376 (12.3)	40 404 (11.8)	2443 (10.3)	8543 (10.8)	6504 (12.3)
4	12 520 (1.2)	3368 (1.0)	575 (2.4)	797 (1.0)	364 (0.7)
5	240 295 (22.0)	74 990 (21.9)	6937 (29.2)	17 032 (21.5)	11 983 (22.7)
6	55 964 (5.1)	12 474 (3.6)	616 (2.6)	2979 (3.8)	2001 (3.8)
7	31 788 (2.9)	6352 (1.9)	530 (2.2)	1239 (1.6)	1023 (1.9)
8	195 366 (17.9)	39 575 (11.5)	2627 (11.1)	6847 (8.7)	6349 (12.0)
Travel time to the nearest VAMC, mean (SD), min	22.65 (24.7)	22.56 (22.5)	21.70 (23.5)	21.58 (22.7)	22.30 (20.3)
Gagne Comorbidity Index score, mean (SD)^c^	0.40 (1.4)	0.53 (1.5)	1.46 (2.2)	0.66 (1.6)	0.59 (1.5)
**VAMCs**					
Academic affiliation	1 065 657 (97.7)	335 851 (97.9)	23 328 (98.1)	77 756 (98.2)	51 765 (97.9)
Unique outpatient visits in fiscal year 2014, mean (SD)	62 708.2 (26 571.5)	63 074.2 (26 676.6)	63 017.7 (26 381.8)	62 947.1 (26 422.4)	60 536.8 (26 679.2)
Census region					
Northeast	145 256 (13.3)	38 934 (11.4)	2918 (12.3)	9119 (11.5)	5739 (10.9)
South	480 550 (44.0)	161 943 (47.2)	10 633 (44.7)	36 803 (46.5)	25 283 (47.8)
Midwest	242 344 (22.2)	70 740 (20.6)	5322 (22.4)	16 838 (21.3)	12 858 (24.3)
West	223 099 (20.4)	71 407 (20.8)	4903 (20.6)	16 416 (20.7)	9009 (17.0)
Complexity rating,^d^					
1a	459 688 (42.1)	144 738 (42.2)	10 525 (44.3)	34 057 (43.0)	20 725 (39.2)
1b	219 750 (20.1)	67 934 (19.8)	4723 (19.9)	16 198 (20.5)	10 176 (19.2)
1c	160 937 (14.75)	52 032 (15.2)	3713 (15.6)	11 574 (14.6)	8927 (16.9)
2	136 168 (12.48)	43 395 (12.7)	2721 (11.4)	9880 (12.5)	7525 (14.2)
3	114 706 (10.51)	34 925 (10.2)	2094 (8.8)	7467 (9.4)	5536 (10.5)

Abbreviation: VAMC, Veterans Affairs medical center.

^a^ Data are presented as number (percentage) of patients or VAMCs unless otherwise indicated.

^b^ Veterans are assigned to a priority group assigned to 1 of 8 priority groups at VA enrollment based on service-connected illnesses, era of service, and socioeconomic status determined by means testing. A veteran’s priority group determines their level of co-payment.

^c^ The Gagne Comorbidity Index is based on 20 conditions derived from the Charlson Comorbidity and Elixhauser indexes and ranged from 2 to 16 in the study cohort.

^d^ The complexity rating is based on a VAMC’s patient volume, number and breadth of physician specialists, patient case mix, intensive care unit capabilities, and degree of teaching and research. VAMCs with a 1a rating are the most complex.

When applying the sensitive criteria, overall and VAMC-level low-value testing frequency varied substantially across conditions: 18.2% (adjusted VAMC range, 10.9%-24.6%) for low back pain, 12.8% (range, 8.6%-22.6%) for headache, 20.1% (range, 16.3%-27.7%) for syncope, and 4.6% (range, 2.7%-10.1%) for sinusitis (Table 3). With the specific criteria, the overall frequency of low-value testing across VAMCs was lower: 5.6% (range, 3.6%-7.7%) for low back pain, 8.6% (range, 6.2%-14.6%) for headache, 13.3% (range, 11.3%-16.8%) for syncope, and 2.4% (range, 1.3%-5.1%) for sinusitis. In veterans with a Gagne Comorbidity Score in the top decile and thus at greatest risk of mortality at 1-year, overall rates and ranges across VAMCs were similar to those demonstrated in the overall cohort when applying both the sensitive and specific criteria. (eTable 1 in the Supplement).

**Table 3.** Receipt of Low-Value Diagnostic Testing by Condition Overall and by VAMC

Medical conditions	Veterans at risk for low-value diagnostic testing, No.^a^	Sensitive criteria				Specific criteria			
		Overall veterans, No. (%)	Unadjusted median veterans per VAMC, %	Unadjusted range across VAMCs, %	Adjusted range across VAMCs, %^b^	Overall veterans, No. (%)	Unadjusted median veterans per VAMC, %	Unadjusted range across VAMCs, %	Adjusted range across VAMCs, %^b^
Nonspecific low back pain	343 024	63 195 (18.4)	18.2	7.5-26.5	10.9-24.6	19 736 (5.8)	5.6	2.8-11.2	3.6-7.7
Uncomplicated headache	79 176	10 518 (13.3)	12.8	1.3-29.0	8.6-22.6	6904 (8.7)	8.6	0.7-19.7	6.2-14.6
Head imaging	NA	10 354 (13.1)	12.7	0.7-28.8	8.4-22.1	6786 (8.6)	8.4	0.0-19.6	6.3-14.3
Electroencephalography	NA	444 (0.6)	0.4	0.0-2.8	0.2-1.5	167 (0.2)	0.1	0.0-1.4	0.1-0.5
Syncope	23 776	4998 (21.0)	20.1	0.0-33.7	16.3-27.7	3300 (13.9)	13.3	0.0-24.2	11.3-16.8
Head imaging	NA	4255 (17.9)	16.9	0.0-32.6	13.1-24.3	2393 (10.1)	9.4	0.0-18.9	7.4-13.0
Carotid ultrasound	NA	1807 (7.6)	6.8	0.0-17.8	4.9-13.6	1390 (5.9)	5.7	0.0-15.4	3.6-9.2
Acute sinusitis	52 889	2583 (4.9)	4.6	0.0-13.9	2.7-10.1	1305 (2.5)	2.4	0.0-7.9	1.3-5.1

Abbreviations: NA, not applicable; VAMC, Veterans Affairs Medical Center.

^a^ Veterans with an *International Classification of Diseases, Ninth Revision* diagnosis code that corresponded to each condition of interest in fiscal year 2014 or before receipt of the related low-value health service in fiscal year 2015.

^b^ VAMC-level frequencies adjusted for the following covariables: age, race/ethnicity, marital status, VA priority group at the time of enrollment, travel time to the nearest VAMC, Gagne Comorbidity Index, variable for VAMC where each veteran received the majority of their outpatient care, academic affiliation, facility size (number of outpatient visits in fiscal year 2014), VAMC complexity rating, and census region.

The median aOR ranged from 1.23 among veterans with low back pain to 1.42 among veterans with acute sinusitis, meaning that the median odds of undergoing low-value testing for low back pain or acute sinusitis increased by 23% or 42%, respectively, if a veteran transferred their care to a VAMC with a higher rate of low-value testing for that service (Table 4). The ICC derived from the unadjusted models, which depicts the proportion of variance in the delivery of low-value testing that was associated with variation between VAMCs, was uniformly small for each disease state, with values ranging from 1.37% (95% CI, 1.06-1.84) for low back pain to 3.99% (95% CI, 2.88-5.89) for acute sinusitis (Table 4). Across all disease states, the ICC was only marginally attenuated by the addition of veteran- and VAMC-level covariates in each model. For both the median aOR and ICC, results were similar when applying the specific criteria (eTable 2 in the Supplement).

**Table 4.** Variation in Low-Value Diagnostic Testing by Condition Defined Using Sensitive Criteria

Parameter	Unadjusted model	Adjusted models	
		Veteran-level covariates only^a^	Veteran- and VAMC-level covariates^a^^,^^b^
Nonspecific low back pain			
OR, median	1.23	1.21	1.21
ICC, % (95% CI)	1.37 (1.06-1.84)	1.25 (0.96-1.69)	1.17 (0.89-1.62)
Syncope			
OR, median	1.25	1.24	1.21
ICC, % (95% CI)	1.63 (1.11-2.62)	1.54 (1.03-2.54)	1.24 (0.79-2.19)
Uncomplicated			
OR, median	1.26	1.24	1.23
ICC, % (95% CI)	1.72 (1.27-2.46)	1.552 (1.13-2.25)	1.405 (1.01-2.09)
Acute sinusitis			
OR, median	1.42	1.39	1.40
ICC, % (95% CI)	3.99 (2.88-5.89)	3.58 (2.54-5.41)	3.62 (2.53-5.59)

Abbreviations: ICC, intraclass correlation coefficient; OR, odds ratio; VAMC, Veterans Affairs medical center.

^a^ Veteran-level covariates included age, race/ethnicity, marital status, VA priority group at the time of enrollment, travel time to the nearest VAMC, and Gagne Comorbidity Index.

^b^ VAMC-level covariates included VAMC where each veteran received the majority of their outpatient care, academic affiliation, facility size (number of outpatient visits in fiscal year 2014), VAMC complexity rating, and census region.

Significant differences were found in the odds of undergoing low-value testing by VAMC decile for each disease state. Among veterans who received care at VAMCs in the top decile for each disease state, the adjusted OR ranged from 2.0 (95% CI, 1.9-2.1) for veterans with low back pain to 4.7 (95% CI, 3.7-6.0) for veterans with acute sinusitis, meaning the odds of undergoing low-value testing for low back pain and sinusitis were 2 and 4.7 times as large, respectively, as the odds of undergoing such testing for veterans who received care at VAMCs in the lowest decile. When applying the specific criteria, differences across deciles were accentuated and ranged from 2.1 (95% CI, 1.9-2.3) for veterans with low back pain to 8.4 (95% CI, 5.5-12.9) for veterans with acute sinusitis (eTables 3 and 4 in the Supplement).

Low-value testing for different conditions was significantly correlated at the VAMC level, with ρ values ranging from 0.18 (*P* = .04) for the correlation between syncope and sinusitis to 0.56 (*P* < .001) for the correlation between syncope and headache (Figure). When evaluating the correlation between the use of separate low-value tests for syncope, head imaging and carotid ultrasonography were significantly correlated when applying both the sensitive (ρ = 0.40; *P* < .001) and specific (ρ = 0.21; *P* = .02) criteria. For headache, use of head imaging and electroencephalography were significantly correlated when applying the sensitive criteria (ρ = 0.26, *P* = .003) but not when applying the specific criteria (ρ = 0.13, *P* = .16).

**Figure.**
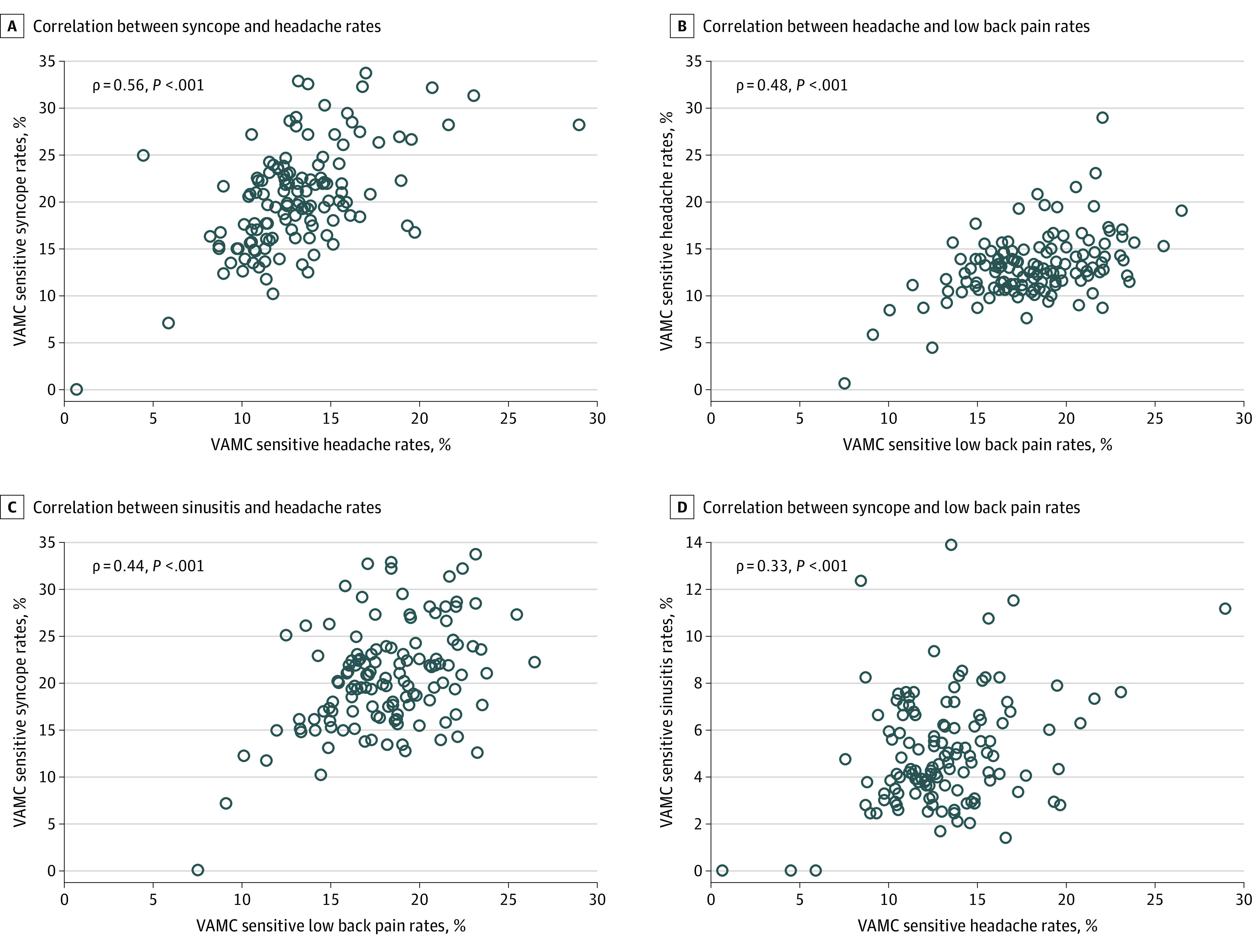
Veterans Affairs Medical Center (VAMC)–Level Correlation Between Low-Value Diagnostic Testing Using Sensitive Criteria

## Discussion

In a national cohort of veterans, low-value diagnostic testing was common, affecting from 5% to 21% of veterans based on their underlying condition and similarly affecting those at greatest risk of 1-year mortality. We also identified 2- to 5-fold variation in low-value testing across VAMCs and significant correlation at the VAMC level in veterans’ receipt of such testing.

Our findings build on prior studies by examining multiple low-value diagnostic tests and expand our understanding of the scope and variability of low-value testing throughout the VHA.^3,16,19,20,29^ Our results align with studies conducted by Rubenstein et al^16^ and Burke et al^19^ that demonstrated overuse and variation of low-value upper endoscopy and neuroimaging throughout the VHA, respectively. The current findings also echo prior work examining low-value prostate cancer screening, which occurred in 17.7% of VA beneficiaries, with 10-fold variation across VAMCs.^18^ In taking a patient-centered approach, we identified low-value testing first by identifying subpopulations of veterans with a corresponding disease state rather than simply those who underwent testing; thus, we depicted our estimates of low-value testing as the frequency of veterans with each condition who received a corresponding low-value test. This strategy differs from prior approaches broadly assessing low-value care among Medicare beneficiaries, which depict low-value care as counts among 100 beneficiaries, making direct comparisons between VA and Medicare beneficiaries challenging.^5,7,8^ Nevertheless, these data, in concert with the growing body of literature on this topic, suggest that low-value care is common and occurs across multiple disease states in the VHA, as it does among Medicare and private insurance beneficiaries.

Our findings are notable in that VA clinicians are not subject to the same incentives and circumstances commonly associated with low-value testing in non-VA settings in the US. For example, unlike VA physicians who are federal employees, non-VA clinicians may be financially incentivized for ordering certain health services, especially if they own the related equipment. VA physicians are also insulated from malpractice lawsuits and have access to an electronic medical record system that contains records from the entirety of the VHA system and incorporates decision-support tools, enhancing the ability of physicians to make clinical decisions.^13,14,15^ Furthermore, the variation that we observed in low-value testing across VAMCs could not be easily explained by the traditional veteran- and VAMC-level covariates that we incorporated in our analyses. This suggests that unique local factors, such as those related to provider culture and variations in the robustness of decision support for ordering tests and procedures at each VAMC, are associated with low-value testing. Similar factors may also play a role in other non-US health systems, such as Canada’s, where low-value care is broadly present despite operating largely within a single payer system.^30^ Identifying these factors will be essential for the development of interventions to reduce the delivery of such care in the VHA and other integrated health care systems.

The correlations of the receipt of low-value testing among the health conditions in our study also suggest that the delivery of such care at VAMCs is associated with systemic factors that are not specific to the delivery of an individual test. Zhou et al^29^ previously developed an index to characterize total systematic health care waste rather than to identify the overuse of specific low-value tests and procedures. They demonstrated that health care systems may be subject to latent overarching pressures to deliver low-value care. To the extent that low-value care delivery is specific to certain tests and procedures, directed interventions and decision-support tools integrated within the electronic medical record system may be sufficient to reduce the delivery of such care. Although the development of additional service-specific interventions is appropriate in certain circumstances, addressing those latent factors associated with the overarching provision of low-value care may require the institution of broad cultural change targeted at the national and VAMC levels. To aid in this task, our claims-based approach to identify low-value testing may help the development of future value-based metrics.^23,30,31,32,33^

### Limitations

This study has limitations. First, value is an inherently subjective construct that cannot be readily detected by administrative claims in every circumstance. By applying both sensitive and specific criteria to identify low-value testing, we demonstrated a range in which the degree of low-value testing likely exists that is sufficient for health systems to triage specific services for interventions. Second, we did not incorporate practitioner characteristics in our models. Such data are not as readily available in the VHA, but in a prior VHA study,^34^ the ratio of nurse practitioners and physician’s assistants to physicians was associated with overuse of low-value prostate-specific antigen testing. This finding shows the importance of characterizing practitioner- and system-level factors both qualitatively and quantitatively in future work. Third, our study only evaluated veterans’ receipt of care in the VHA. Nearly all veterans 65 years or older are dually enrolled in the VHA and Medicare, and others are increasingly receiving non-VA care that is paid for by the VHA.^15,31,35^ A greater understanding of veterans’ broad receipt of low-value care across all health care systems is needed to enhance the overall value of care that they receive.

## Conclusions

In this cohort study, low-value diagnostic testing for 4 conditions was common and variably delivered throughout the VHA despite the VHA being a non–fee-for-service integrated health care system. Our findings suggest the need to further characterize the unique factors associated with the overarching delivery of low-value care in the VHA to ensure that veterans may make informed health care choices and that the VHA is poised to be a model health care system in ensuring that its beneficiaries receive high-value care that is directly delivered or is paid for by the VHA.

## Notes

Supplement. **eAppendix.** **eTable 1.** Receipt of Low-Value Diagnostic Testing by Condition Overall and by VA Medical Center among Veterans at Greatest Risk of Mortality at 1 Year **eTable 2. **Variation in Low-Value Diagnostic Testing by Condition Defined Using Specific Criteria **eTable 3. **Frequency and Adjusted Odds Ratio of Low-Value Diagnostic Testing by Condition and VAMC decile of Low-Value Diagnostic Testing Using Sensitive Criteria **eTable 4. **Frequency and Adjusted Odds Ratio of Low-Value Diagnostic Testing by Condition and VAMC decile of Low-Value Diagnostic Testing Using Specific Criteria
