# Supplementary material for: Evaluation of Low-Value Diagnostic Testing for 4 Common Conditions in the Veterans Health Administration
Source: JAMA Netw Open. 2020 Sep 22;3(9):e2016445. doi: 10.1001/jamanetworkopen.2020.16445 (PMC7509631; doi:10.1001/jamanetworkopen.2020.16445)
Supplement: Supplement. — eAppendix. eTable 1. Receipt of Low-Value Diagnostic Testing by Condition Overall and by VA Medical Center among Veterans at Greatest Risk of Mortality at 1 Year eTable 2. Variation in Low-Value Diagnostic Testing by Condition Defined Using Specific Criteria eTable 3. Frequency and Adjusted Odds Ratio of Low-Value Diagnostic Testing by Condition and VAMC decile of Low-Value Diagnostic Testing Using Sensitive Criteria eTable 4. Frequency and Adjusted Odds Ratio of Low-Value Diagnostic Testing by Condition and VAMC decile of Low-Value Diagnostic Testing Using Specific Criteria [file jamanetwopen-e2016445-s001.pdf]

Supplementary Online Content

Radomski TR, Feldman R, Huang Y, et al. Evaluation of low-value diagnostic testing for 4 common conditions in the Veterans Health Administration. *JAMA Netw Open*. 2020;3(9):e2016445. doi:10.1001/jamanetworkopen.2020.16445

eAppendix.

eTable 1. Receipt of Low-Value Diagnostic Testing by Condition Overall and by VA Medical Center among Veterans at Greatest Risk of Mortality at 1 Year

eTable 2. Variation in Low-Value Diagnostic Testing by Condition Defined Using Specific Criteria

eTable 3. Frequency and Adjusted Odds Ratio of Low-Value Diagnostic Testing by Condition and VAMC decile of Low-Value Diagnostic Testing Using Sensitive Criteria

eTable 4. Frequency and Adjusted Odds Ratio of Low-Value Diagnostic Testing by Condition and VAMC decile of Low-Value Diagnostic Testing Using Specific Criteria

This supplementary material has been provided by the authors to give readers additional information about their work.

© 2020 Radomski TR et al. *JAMA Network Open.*

eAppendix.
Low-Value Diagnostic Test: Back imaging for patients with non-specific low back pain

Denominator: Patients with a back pain diagnosis:

- **Back Pain ICD-9 Codes**: 7213 72190 72210 72252 7226 72293 72402 7242-7246 72470 72471 72479 7385 7393 7394 8460-8463 8468 8469 8472

Sensitive: Patient who underwent back imaging with a low back pain diagnosis 6 weeks prior to testing

- **Back imaging CPT Codes**: 72010 72020 72052 72100 72110 72114 72120 72200 72202 72220 72131-72133 72141 72142 72146-72149 72156 72157 72158 (radiologic, CT, and MRI imaging of spine)

Specific: Exclude patients with a specific diagnosis that occurred within 30 days prior to the date of the back imaging test

- **ICD-9 Codes**: 140xx–208xx 230xx-239xx (cancer), 800x-839xx 850xx-854xx 86xxx 905xx-909xx 92611 92612 929, 952xx 958xx-959xx (trauma), 3040x-3042x 3044x 3054x-3057x (IV drug abuse), 34460 7292x (neurologic impairment), 4210 4211 4219 (endocarditis), 038xx (septicemia), 01xxx (tuberculosis), 730xx (osteomyelitis), 7806x 7830x 7832x 78079 7808x 2859x (fever, weight loss, malaise, night sweats, anemia not due to blood loss)

OR

- Exclude patients if imaging occurred greater than 6 weeks after first diagnosis of back pain

© 2020 Radomski TR et al. *JAMA Network Open.*

<u>Low-Value Diagnostic Test:</u> Head imaging for uncomplicated headache

<u>Denominator</u>: Patients with a headache diagnosis

- Headache diagnosis codes: 30781, 339xx , 364x, 7840 (headache or migraine)

<u>Sensitive</u>: Any patient who underwent imaging who had a headache diagnosis 30 days prior to imaging

- CPT codes for head imaging (CT or MRI): 70450, 70460, 70470, 70551-70553 (CT or MRI of head or brain)

<u>Specific:</u> Exclude patients with a specific diagnosis warranting head imaging that was also coded within 30 days prior to head imaging:

- 33920-33922, 33943 (post-traumatic or thunderclap headache); 140xx–208xx, 230xx-239xx (cancer); 3463x , 3466x (migraine with hemiplegia or infarction); 4465 (giant cell arteritis); 345xx 7803x (epilepsy or convulsions); 43xx (cerebrovascular diseases, including stroke/TIA and subarachnoid hemorrhage); 800xx-804xx, 850xx-854xx, 870xx-873xx, 9590x, 910xx, 920xx-921xx (head or face trauma); 78097, 781xx, 7845x (altered mental status, nervous and musculoskeletal system symptoms, including gait abnormality, meningismus, disturbed skin sensation, speech deficits); V1254, V10xx (personal history of stroke/TIA or cancer)

Low-Value Diagnostic Test: Electroencephalogram (EEG) for headaches

Denominator: Patients with a headache diagnosis

- ICD-9 Diagnosis Codes for Headache: 30781, 339xx, 346x, 7840

Sensitive: Patients who underwent EEG in FY2015 with a headache diagnosis in the past 30 days

- CPT codes for EEG: 95812, 95813, 95816, 95819, 95822, 95827, 95830, 95957

Specific: Exclude patients with a history of epilepsy or convulsion at any time in FY 2014-2015

- ICD-9 codes: 345xx, 7803x, 7810

© 2020 Radomski TR et al. *JAMA Network Open.*

Low-Value Diagnostic Test: Head imaging in patients with syncope (passing out)

Denominator: Patients with a syncope diagnosis

- ICD-9 Diagnosis Codes for Syncope: 7802, 9921

Sensitive: Patients who underwent head imaging with a syncope diagnosis within 30 days prior to testing

- CPT codes for head imaging: 70450, 70460, 70470, 70551-70553 (CT or MRI of head or brain)

Specific: Exclude patients with a diagnosis within 30 days prior to the date of the head imaging test :

- ICD-9 codes: 345xx, 7803x (epilepsy or convulsions); 43xx (cerebrovascular diseases, including stroke/TIA and subarachnoid hemorrhage); 800xx-804xx, 850xx-854xx, 870xx-873xx, 9590x, 910xx, 920xx-921xx, (head or face trauma); 78097, 781xx, 7820, 7845x, (altered mental status, nervous and musculoskeletal system symptoms, including gait abnormality, meningismus, disturbed skin sensation, speech deficits); V1254, V10xx (personal history of stroke/TIA)

© 2020 Radomski TR et al. *JAMA Network Open.*

Low-Value Diagnostic Test: Assessing for carotid artery disease in patients with syncope (passing out)

Denominator: Patients with a syncope diagnosis

- ICD-9 Diagnosis Codes for Syncope: 7802, 9921

Sensitive: Patients who underwent carotid imaging with a syncope diagnosis within 30 days prior to testing

- CPT codes for carotid artery imaging: 36222-36224, 70498, 70547-70549, 93880, 93882, 3100F

Specific: Exclude patients with a diagnosis of stroke, transient ischemic attack (TIA) or focal neurologic symptoms that occurred within 30 days prior to testing:

- ICD-9 codes for stroke, TIA, focal neurologic symptoms: 430, 431, 43301, 43311, 43321, 43331, 43381, 43391, 43400, 43401, 43410, 43411, 43490, 43491, 4350, 4351, 4353, 4358, 4359, 436, 99702, V1254, (stroke/TIA); 3623, 36284 (retinal vascular occlusion/ischemia); 781xx, 7820, 78451, 78452, 78459 (nervous and musculoskeletal symptoms)

© 2020 Radomski TR et al. *JAMA Network Open.*

<u>Low-Value Diagnostic Test:</u> CT Scan of the sinuses for uncomplicated acute rhinosinusitis

<u>Denominator</u>: Patients with Sinusitis

- ICD-9 Diagnosis Codes for Sinusitis: 461x, 473x

<u>Sensitive</u>: Patients who underwent CT of the sinuses in FY2015 with a diagnosis of sinusitis **within 30 days prior to the CT Scan**

- CPT codes for CT Scan of the sinuses: 70486, 70487,70488

<u>Specific</u>: Exclude patients with another diagnosis that would warrant imaging coded within the past **30 days**

- ICD-9 codes: 2770x, 042xx, 07953, 279xx (immune disorders); 471x (nasal polyp), 373xx, 37600 (eyelid/orbit inflammation); 800xx-804xx, 850xx-854xx, 870xx-873xx 9590x, 910xx, 920xx, 921xx (head or face trauma)

OR

- Exclude patients who have a diagnosis of sinusitis (codes 461x, 473x) 30-365 days prior to undergoing the CT Scan, as this suggests chronic sinusitis

© 2020 Radomski TR et al. *JAMA Network Open.*

**eTable 1:** Receipt of Low-Value Diagnostic Testing by Condition Overall and by VA Medical Center among Veterans at Greatest Risk of Mortality at 1 Year[a]									
**Medical Conditions**	**Veterans at Risk for Low-Value Diagnostic Testing[b]**	**Sensitive Algorithm**				**Specific Algorithm**			
		Veterans Overall, N (%)	Unadjusted Median Veterans per VAMC, (%)	Unadjusted Range (min – max) across VAMCs, (%)	Adjusted Range (min – max) across VAMCs, (%)	Veterans Overall, N (%)	Unadjusted Median Veterans per VAMC, (%)	Unadjusted Range (min – max) across VAMCs, (%)	Adjusted Range (min – max) across VAMCs, (%)
Non-specific low back pain	28,217 (8.2)	4,779 (16.9)	16.9	5.5 – 25.0	14.5 – 19.1	846 (3.0)	2.9	0 – 9.7	2.4 – 3.3
Uncomplicated headache	7,346 (9.3)	1,263 (17.2)	16.1	3.8 – 43.8	12.2 – 21.9	526 (7.2)	6.9	0 – 30.8	5.2 – 12.3
Head imaging	-	1,251 (17.0)	16.0	3.8-43.8	12.1 – 21.7	513 (7.0)	6.3	0 – 30.8	4.6 – 12.6
Electroencephalogram	-	58 (0.8)	0	0 – 6.3	0.2 – 1.3	17 (0.2)	0	0 – 4.8	0.1 – 0.5
Syncope	2,364 (9.9)	461 (19.5)	17.6	0 – 66.7	14.7 – 25.5	245 (10.4)	8.8	0 – 66.7	6.7 – 14.0
Head imaging	-	410 (17.3)	15.4	0 – 40.0	12.4 – 23.8	188 (8.0)	6.2	0 – 33.3	6.2 – 8.6
Carotid ultrasound	-	137 (5.8)	4.0	0 – 40.0	4.9 – 6.4	91 (3.9)	0	0 – 40.0	3.1 – 3.1
Acute Sinusitis	4,715 (8.9)	214 (4.5)	3.8	0 – 33.3	1.8 – 8.3	101 (2.1)	0	0 – 22.2	0.7 – 7.0

Abbreviations: VAMC, Veterans Affairs Medical Center

a. Veterans included are those with a Gagne Score in the top decile of the overall cohort

b. Veterans with an ICD-9 diagnosis code that corresponded to each conditions of interest in FY 14 or prior to receipt of the related low-value health service in FY15

**eTable 2:** Variation in Low-Value Diagnostic Testing by Condition Defined Using Specific Criteria

© 2020 Radomski TR et al. *JAMA Network Open.*

Parameters by Condition	Unadjusted Model	Adjusted Models	
		Veteran-level Covariates only[a]	Veteran and VAMC-level covariates[a,b]
**Uncomplicated Low-Back Pain**			
Odds Ratio, median	1.22	1.20	1.19
ICC, % (95% CI)	1.27 (0.96, 1.76)	1.07 (0.79, 1.52)	1.0 (0.72, 1.46)
**Syncope**			
Odds Ratio, median	1.22	1.23	1.20
ICC, % (95% CI)	1.36 (0.86, 2.45)	1.40 (0.88, 2.55)	1.13 (0.67, 2.31)
**Uncomplicated Headache**			
Odds Ratio, median	1.25	1.24	1.22
ICC, % (95% CI)	1.68 (1.20, 2.53)	1.57 (1.11, 2.41)	1.35 (0.93, 2.16)
**Acute Sinusitis**			
Odds Ratio, median	1.49	1.46	1.42
ICC, % (95% CI)	5.12 (3.58, 7.88)	4.58 (3.13, 7.29)	4.04 (2.65, 6.86)

Abbreviations: ICC, Intraclass correlation coefficient
a. Veteran-level Covariates: age, race/ethnicity, marital status, VA priority group at the time of enrollment, travel time to the nearest VAMC, and Gagne Comorbidity Score.
b. VAMC-level Covariaties: VAMC where each Veteran receives the majority of their outpatient care, academic affiliation, facility size (depicted by number of outpatient visits in FY14), VAMC complexity rating, and Census Region Location.

© 2020 Radomski TR et al. *JAMA Network Open.*

eTable 3: Frequency and Adjusted Odds Ratio of Low-Value Diagnostic Testing by Condition and VAMC decile of Low-Value Diagnostic Testing Using Sensitive Criteria

Decile	Uncomplicated Low Back Pain (N = 343,024)		Syncope (N = 23,776)		Uncomplicated Headache (N = 79,176)		Acute Sinusitis (N = 52,889)	
	Frequency (%)[a]	Adjusted OR (95% CI)[b]	Frequency (%)[a]	Adjusted OR (95% CI)[b]	Frequency (%)[a]	Adjusted OR (95% CI)[b]	Frequency (%)[a]	Adjusted OR (95% CI)[b]
1	13.1	Reference	12.2	Reference	8.5	Reference	2.1	Reference
2	15.2	1.2 (1.1, 1.3)	15.2	1.3 (1.0, 1.6)	10.4	1.2 (1.1, 1.4)	2.9	1.3 (1.0, 1.7)
3	16.2	1.3 (1.3, 1.4)	16.8	1.4 (1.2, 1.8)	11.1	1.4 (1.2, 1.5)	3.5	1.6 (1.3, 2.1)
4	16.9	1.4 (1.3, 1.5)	18.3	1.6 (1.3, 2.0)	12.0	1.4 (1.3, 1.6)	4.0	1.8 (1.4, 2.3)
5	17.8	1.5 (1.4, 1.6)	19.8	1.8 (1.5, 2.3)	12.6	1.5 (1.3, 1.7)	4.4	2.1 (1.6, 2.6)
6	18.6	1.5 (1.5, 1.6)	21.4	2.0 (1.6, 2.4)	13.2	1.6 (1.4, 1.8)	5.0	2.4 (1.9, 3.0)
7	19.4	1.6 (1.5, 1.7)	22.2	2.0 (1.6, 2.5)	14.0	1.8 (1.6, 2.0)	5.8	2.8 (2.2, 3.5)
8	20.8	1.7 (1.6, 1.8)	23.8	2.2 (1.8, 2.7)	14.9	1.9 (1.7, 2.1)	6.6	3.1 (2.5, 3.9)
9	21.8	1.8 (1.8, 2.0)	27.0	2.6 (2.1, 3.2)	16.0	2.0 (1.8, 2.3)	7.5	3.6 (2.8, 4.6)
10	23.6	2.0 (1.9, 2.1)	30.7	3.1 (2.5, 3.9)	20.7	2.6 (2.3, 3.0)	9.6	4.7 (3.7, 6.0)

a. Frequency depict the unadjusted mean rates of VAMCs within the corresponding decile.
b. Odds ratios adjusted for the following patient-level and VAMC-level factors Patient-level factors (FY2014): age, race/ethnicity, marital status, VA priority group at the time of enrollment, travel time to the nearest VAMC and Gagne Comorbidity Score. VAMC factors: VAMC where each Veteran receives the majority of their outpatient care, academic affiliation, facility size (depicted by number of outpatient visits in FY14), VAMC complexity rating, and Census Region Location.
c. Abbreviations: VHA = Veterans Health Administration, VAMC = VA Medical Center, OR = Odds Ratio

© 2020 Radomski TR et al. *JAMA Network Open.*

eTable 4: Frequency and Adjusted Odds Ratio of Low-Value Diagnostic Testing by Condition and VAMC decile of Low-Value Diagnostic Testing Using Specific Criteria

Decile	Uncomplicated Low Back Pain (N = 343,024)		Syncope (N = 23,776)		Uncomplicated Headache (N = 79,176)		Acute Sinusitis (N = 52,889)	
	Frequency (%)[a]	Frequency OR (95% CI)[b]	Frequency (%)[a]	Adjusted OR (95% CI)[b]	Frequency (%)[a]	Adjusted OR (95% CI)[b]	Frequency (%)[a]	Adjusted OR (95% CI)[b]
1	3.9	Reference	7.9	Reference	4.7	Reference	0.7	Reference
2	4.4	1.2 (1.1, 1.3)	9.7	1.3 (1.0, 1.6)	6.1	1.3 (1.0, 1.5)	1.2	1.8 (1.1, 2.8)
3	4.8	1.23 (1.2, 1.4)	11.0	1.5 (1.1, 1.9)	7.2	1.5 (1.3, 1.8)	1.5	2.3 (1.5, 3.6)
4	5.2	1.4 (1.3, 1.6)	11.9	1.6 (1.3, 2.0)	7.8	1.7 (1.4, 2.0)	1.9	2.6 (1.7, 4.0)
5	5.5	1.5 (1.4, 1.6)	12.9	1.7 (1.4, 2.2)	8.4	1.8 (1.5, 2.1)	2.3	3.3 (2.1, 5.0)
6	5.8	1.6 (1.4, 1.7)	14.0	1.9 (1.5, 2.4)	8.8	1.9 (1.6, 2.3)	2.6	3.9 (2.6, 5.8)
7	6.1	1.6 (1.4, 1.7)	15.0	2.1 (1.6, 2.6)	9.5	2.0 (1.7, 2.4)	3.1	4.6 (3.0, 6.9)
8	6.6	1.7 (1.6, 1.9)	16.2	2.3 (1.8, 2.9)	10.2	2.1 (1.7, 2.5)	3.4	4.9 (3.2, 7.5)
9	7.0	1.8 (1.7, 2.0)	18.6	2.7 (2.1, 3.5)	10.9	2.4 (2.0, 2.9)	4.1	6.0 (3.9, 9.2)
10	8.0	2.1 (1.9, 2.3)	21.1	3.2 (2.5, 4.0)	13.3	3.0 (2.5, 3.6)	5.8	8.4 (5.5, 12.9)

a. Frequency depict the unadjusted mean rates of VAMCs within the corresponding decile.
b. Odds ratios adjusted for the following patient-level and VAMC-level factors Patient-level factors (FY2014): age, race/ethnicity, marital status, VA priority group at the time of enrollment, travel time to the nearest VAMC and Gagne Comorbidity Score. VAMC factors: VAMC where each Veteran receives the majority of their outpatient care, academic affiliation, facility size (depicted by number of outpatient visits in FY14), VAMC complexity rating, and Census Region Location.
c. Abbreviations: VHA = Veterans Health Administration, VAMC = VA Medical Center, OR = Odds Ratio

© 2020 Radomski TR et al. *JAMA Network Open.*
